# Outcomes of endoscopic management of primary and refractory postcholecystectomy biliary leaks in a multicentre review of 178 patients

**DOI:** 10.1186/s12876-015-0334-y

**Published:** 2015-08-19

**Authors:** Jorge Canena, David Horta, João Coimbra, Liliane Meireles, Pedro Russo, Inês Marques, Leonel Ricardo, Catarina Rodrigues, Tiago Capela, Diana Carvalho, Rafaela Loureiro, António Mateus Dias, Gonçalo Ramos, António Pereira Coutinho, Carlos Romão, Pedro Mota Veiga

**Affiliations:** 1 Department of Gastroenterology, Doutor Fernando Fonseca Hospital, IC 19, 2720-276 Amadora, Portugal; 2 Department of Gastroenterology, Pulido Valente Hospital at Centro Hospitalar Lisboa Norte, Alameda das Linhas de Torres n° 117, 1769-001 Lisbon, Portugal; 3 Department of Gastroenterology, Santo António dos Capuchos Hospital at Centro Hospitalar Lisboa Central, Nova Medical School-Faculty of Medical Sciences, Alameda Santo António dos Capuchos, 1169-050 Lisbon, Portugal; 4 Department of Endoscopy, José Joaquim Fernandes Hospital at Unidade Local de Saúde do Baixo Alentejo, Rua Doutor António Fernandes Covas Lima, 7800-309 Beja, Portugal; 5 Curva de Gauss – Research, Training and Consulting, Rua Doutor Eduardo Maria dos Santos, Lote 1, Loja 3, 3525-000 Canas de Senhorim, Portugal

## Abstract

**Background:**

Biliary leaks have been treated with endoscopic management using different techniques with conflicting results. Furthermore the appropriate rescue therapy for refractory leaks has not been established. We evaluated the clinical effectiveness of initial endotherapy for postcholecystectomy biliary leaks using an homogenous approach (sphincterotomy + placement of a 10-French plastic stent) in a large series of patients as well as the optimal and efficacy of rescue endotherapy for refractory biliary leaks.

**Methods:**

This was a multicenter, retrospective study of 178 patients who underwent endoscopic management of postcholecystectomy biliary leaks with a combination of biliary sphincterotomy and the placement of a large-bore (10-French) plastic stent. Data were collected to analyze the clinical outcomes and technical success, efficacy of the rescue endotherapy and the need for surgery, adverse events and prognostic factors for clinical success of endotherapy.

**Results:**

Following endotherapy, closure of the leak was accomplished in 162/178 patients (91.0 %). The multivariate logistic model showed that the type of leak, namely a high-grade biliary leak, was the only independent prognostic factor associated with treatment failure (OR = 26.78; 95 % CI = 6.59–108.83; *P* < 0.01). The remaining 16 patients were treated with multiple plastic stents (MPSs) with a success rate of 62.5 % (10 patients). The use of fewer than 3 plastic stents (*P* = 0.023) and a high-grade biliary leak (*P* = 0.034) were shown to be significant predictors of treatment failure with MPSs in refractory bile leaks. The 6 patients in whom the placement of MPSs failed were retreated with a fully cover self-expandable metallic stent (FCSEMS), resulting in closure of the leak in all cases.

**Conclusions:**

Endotherapy of biliary leaks with a combination of biliary sphincterotomy and the placement of a large-bore plastic stent is associated with a high rate of success (90 %). However in our series there were several failures using MPSs as a strategy for rescue endotherapy suggesting that refractory biliary leaks should be treated with FCSEMS especially in patients with high-grade leaks.

## Background

Iatrogenic injury to the biliary tree, namely biliary leaks, can occur after laparoscopic cholecystectomy in up to 1 % of cases [1, 2]. In the early years of treatment and in several published case series, surgery was the primary treatment for such cases [2–4]. However, the treatment of bile leaks has evolved into a minimally invasive procedural technique (i.e., endoscopic retrograde cholangiopancreatography [ERCP]) in which decreasing or eliminating the pressure gradient between the bile duct and the duodenum permits preferential transpapillary bile flow instead of extravasation through the leak [1–3, 5–12]. Although the widespread use of ERCP has been shown to be safe and efficacious, leak closure can be accomplished by different endoscopic interventions, and the optimal endoscopic intervention has not established [5, 8]. Biliary stenting or nasobiliary drainage, with or without sphincterotomy, or biliary sphincterotomy alone can be used to treat post-operative bile leaks, with similar results [1–3, 5–11]. One group proposed classifying the severity and treatment of leaks into low grade and high grade, suggesting that complex leaks and high-grade leaks are better treated with a combination of biliary sphincterotomy and transpapillary biliary stent placement [3]. However, there have been reports of leaks being managed with several endoscopic procedures or even endoscopic treatment failures, in which operative therapy is required [1, 3, 6, 11]. Clear strategies for treating these refractory biliary leaks have not emerged, and treatment is provided on an individual basis [13–16]. Currently, two types of endoscopic treatments have been used as an effective rescue therapy for refractory biliary leaks: 1) the temporary placement of a fully covered self-expandable metal stent (FCSEMS) [13–21], and 2) treatment with more than one plastic stent, which can theoretically manage the refractory bile leak at a lower cost [13–16]. Despite a large body of data supporting the use of ERCP in this setting, there has been no study of a large series of patients treated using standard and homogeneous approaches to maximize the benefits of the first endoscopic treatment, a combination of biliary sphincterotomy and the placement of a large-bore (10-French) plastic stent. Furthermore, it is unclear which type of rescue endotherapy should be used after failure of the initial treatment. It is also unclear which prognostic factors are associated with the failure of the initial treatment and the available rescue endotherapy. Therefore, we conducted a retrospective study of a large series of patients, with the aim of evaluating the clinical effectiveness of initial endotherapy for postcholecystectomy biliary leaks, as well as the optimal and efficacy of rescue endotherapy for refractory biliary leaks. Additionally, we evaluated the prognostic factors associated with clinical success of the initial treatment and different types of rescue endotherapy.

## Methods

### Patients and settings

In this multicentre, single-arm, retrospective clinical study, interventional endoscopy database records from 4 institutions were retrospectively reviewed to identify all consecutive patients underwent endoscopic management for postcholecystectomy biliary leaks between March 2009 and July 2014. The following criteria were used for inclusion in this study: 1) initial treatment of the biliary leak with a combination of biliary sphincterotomy and the placement of a 10-French transpapillary biliary stent; 2) after failure of the initial treatment, the patients were subjected to a new endotherapy with multiple plastic stents (MPS); and 3) after pacing the MPS, patients with a persistent biliary leak were submitted to the temporary placement of an FCSEMS, and the patients for whom the placement of an FCSEMS did not close the leak were considered for surgery. Patients with refractory bile leaks with aetiology other than postcholecystectomy were excluded from the study.

Data were collected from various sources, including patients’ charts (manual and electronic), endoscopic and radiology reports, clinical notes, our prospective database, follow-up clinic visits and structured telephone interviews with the treating physicians, patients or family during follow-up or at the time of manuscript preparation. The collected data included patient demographics, anatomical location and type of biliary leak, technical success, treatment outcomes, type of stents used, need for reintervention and adverse events. This study was conducted at 4 institutions (2 tertiary referral academic centres and 2 general district hospitals). All patients provided written informed consent prior to undergoing any procedures. Each of the institutional review boards involved in this study approved this research (Ethics committee of Amadora-Sintra Hospital, ethics committee of Pulido Valente Hospital-Centro Hospitalar Lisboa Norte, ethics committee of Capuchos Hospital-Centro Hospitalar Lisboa Central and ethics committee of Beja Hospital-Unidade Local de Saúde do Baixo Alentejo).

### Endpoints and definitions

The primary endpoint was the clinical success of the endoscopic management of the biliary leaks, for the initial and subsequent rescue types of endotherapy, which was defined as closure of the leak. The secondary endpoints included technical success, potential risk factors associated with the leak closure, treatment duration, adverse events and the need for surgery. After failure of the initial endotherapy, the bile leaks were considered to be refractory biliary leaks, which were defined as leaks that failed to close after endoscopic intervention with a combination of biliary sphincterotomy and the placement of a 10-French transpapillary biliary stent, regardless of the biliary leak location (e.g., cystic stump, common bile duct/common hepatic duct, Luschka’s joints) [14]. High-grade biliary leaks were defined as leaks that were observed fluoroscopically prior to intrahepatic opacification [3]. In cases in which the bile leak was associated with bile duct injury, the Amsterdam Classification was used [22]. Leak closure was considered after the cessation of bile output, which was defined as biliary drainage of less than 5 ml/day in the percutaneous drains and was confirmed at the follow-up ERCP [14]. Endotherapy failure was defined as persistent biliary drainage through the percutaneous drain or as a persistent bile leak at follow-up ERCP. Reintervention was defined as the need for further intervention to control the leak after the initial endotherapy for the refractory leak, including repeat ERCP for additional stenting or surgery. Adverse events were defined as any adverse event related to the ERCP or stent placement, and adverse events were carefully monitored using previously determined definitions [23].

### Technique, stents and follow-up

All ERCP procedures were performed with patients placed in the prone position under sedation with propofol administered by the anaesthesia staff. All procedures were performed or supervised by 3 experienced pancreatobiliary endoscopists (the three first authors). During the first ERCP procedure, a cholangiography was obtained to document evidence of biliary leakage, as well as the site and grade of the bile leak and to determine whether there was an associated major injury of the bile duct, namely a bile duct stricture resulting from direct surgical trauma. After documenting the leak, the endoscopist used a standard protocol that has been used for the last 8 years at the institutions where the study was conducted, specifically a combination of biliary sphincterotomy and the placement of a 10-French transpapillary biliary stent. All plastic stents used were at least 7 cm long, although the plastic stent was not routinely inserted above the leak site. For this treatment, polyethylene stents were used in all cases (Advanix Biliary Stent, Boston Scientific, Natick, MA, USA; Cotton-Huibregtse biliary stent, Cook Medical, Winston-Salem, NC, USA). All patients included in the study were routinely treated with a percutaneous drain and monitored, and the drainage output was used to evaluate the success of endotherapy in closing the biliary leak. After clinical evidence of complete resolution of the bile leak, when appropriate, the patients were scheduled for a second ERCP to remove the plastic stent, and a new cholangiogram was obtained to document the leak closure. However, the patients with similar or increased levels of percutaneous drainage and maintenance or worsening bilious fluid collection on an abdominal ultrasonography or a CT scan after first ERCP were considered to have a refractory bile leak and underwent second ERCP. After confirming a persistent bile leak, the patients underwent more than one plastic stent placement. For this treatment, the endoscopist determined the number and diameter of the plastic stents to be inserted based on the diameter of the bile duct itself. After the MPS treatment, the patients with persistent percutaneous drainage were again considered to be treatment failures and were submitted to a third ERCP in which the plastic stents were removed and a new cholangiogram was performed. Patients with persistent biliary leakage were considered for the placement of a FCSEMS. For this type of endotherapy, an FCSEMS with a 10 mm luminal diameter and a length that varied from 40 to 80 mm was used to allow the stent to be placed above the leak site in all cases. For endotherapy with a FCSEMS, two types of stents were used: the WallFlex (Boston Scientific, Natick, Massachusetts, USA) and the Niti-S (TaeWoong Medical, Seoul, South Korea). Patients for whom the temporary placement of a FCSEMS failed to close the leak were evaluated for surgery.

The follow-up began at the end of the treatment and leak closure and lasted for three months. Three months after hospital discharge, the patients were evaluated by physical examination, blood samples and upper abdominal ultrasonography. The follow-up was performed by reviewing the clinical notes of the treating physician, as well as by reviewing the patients’ manual and electronic charts.

### Statistical analysis

The intention-to-treat method was used for all analyses. The cumulative leak-free survival (clinical success) was evaluated using the Kaplan-Meier method, and the 95 % confidence interval (CI) for long-term leak-free survival was calculated using the Clopper-Pearson (exact) method. The potential risk factors associated with closing the leak were assessed using univariate analysis, followed by multiple logistic regression analysis, as appropriated. The univariate analysis was conducted using the *χ*2 test, with the Monte Carlo simulation (as needed) for categorical variables and the Mann-Whitney *U* test and Student’s *t* test for continuous variables. All reported P values were for a two-tailed test, and *P* < 0.05 was considered to be statistically significant. All statistical analyses were performed using the SPSS software package, version 22 (Statistical Package for the Social Sciences, IBM Corporation, Armonk, NY, USA).

## Results

### Patients

In total, 178 patients (85 males and 93 females) with a median age of 67 years (range, 23-92 years) were enrolled in the study. Fourteen patients (7.9 %) required conversion of laparoscopic cholecystectomy to an open procedure. Patients were referred to ERCP after a median of 7 days (range, 1-28 days) if they had a clinical suspicion of a postcholecystectomy biliary complication based on symptoms (e.g., abdominal pain), abnormal liver-associated enzymes or jaundice, imaging studies (e.g., CT scan, upper abdominal ultrasonography) or biliary leakage from the drains placed during surgery. The most common bile leakage site was the cystic duct stump, which was observed in 109 patients (61.2 %). Twenty-seven patients (15.2 %) had a biliary leak located at the common bile duct/common hepatic duct, which was associated with a bile duct injury, namely a bile duct stricture (Amsterdam type B injury). In total, high-grade biliary leaks were found in 19 patients (10.7 %). Of the 178 patients with a post-operative bile leak, 33 (18.5 %) patients had associated retained common bile duct stones. Patient baseline data are summarized in Table 1.

**Table 1:** Baseline patient data

Characteristic	
Gender	
Male, n (%)	85 (47.8)
Female, n (%)	93 (52.2)
Age (years), median (range; mean)	67 (23–92; 63.8)
Indications for cholecystectomy	
Acute cholecystitis, n (%)	50 (28.1)
Chronic cholecystitis, n (%)	28 (15.7)
Symptomatic cholelithiasis, n (%)	100 (56.2)
Type of surgery	
Laparoscopic cholecystectomy, n (%)	171 (96.1)
Laparoscopic cholecystectomy with conversion, n (%)	7 (3.9)
Site of bile leak, n (%)	
Cystic duct stump	109 (61.2)
CBD/CHD	27 (15.2)
Luschka	42 (23.6)
Type of leak, n (%)	
High-grade leak	19 (10.7)
Low-grade leak	159 (89.3)
Associated biliary stricture, n (%)	
Yes	27 (15.2)
No	151 (84.8)
Time to first ERCP (first biliary stenting) after cholecystectomy (days), median (range; mean)	7 (1–28; 7.5)

*CBD/CHD* common bile duct/common hepatic duct

### Characteristics of the first endoscopic treatment, leak-free survival and prognostic factors associated with closure of the leak

**Fig. 1:**
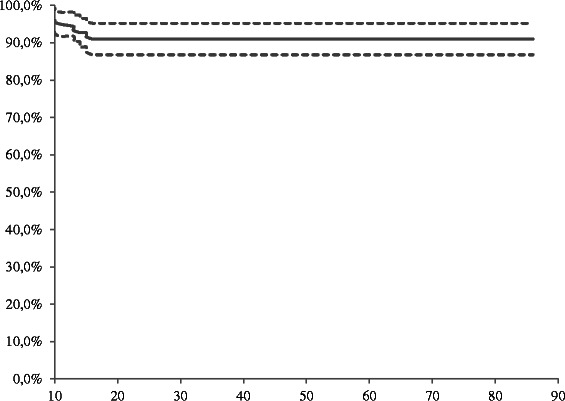
Kaplan-Meier analysis of leak-free survival rates (clinical success) after first endoscopic treatment for cholecystectomy biliary leaks with a combination of biliary sphincterotomy and the placement of a 10-French plastic stent (solid line) and 95 % CI (dashed line)

Stent implantation was technically successful in all patients. The initial endoscopic management of the 178 patients with biliary leaks is shown in Table 2. Regarding the clinical success of the first endotherapy, a total of 162 of 178 patients (91.0 %) who underwent temporary placement of a 10-French plastic stent and a biliary sphincterotomy had their bile leaks closed. In the 162 clinically successful cases, bile output ceased in the percutaneous drains after a median time of 6 days (range, 2–9 days), and the plastic stents were removed after a median period of 67 days (range, 30–67 days). After the initial endoscopic treatment, the Kaplan-Meier analysis showed that the estimated cumulative mean time of clinical success was 50.7 days (95 % CI: 47.5–53.8 days). By the end of the initial endotherapy, the leak-free survival rate was 91.0 % (95 % CI: 86.9–95.1 %) at 30 and 60 days (Fig. 1). The univariate analysis of treatment failure with a combination of biliary sphincterotomy and the placement of a 10-French biliary stent is shown in Table 3. Of the 5 evaluated variables (i.e., sex, age, biliary leak location, type of leak, and interval between the surgery and the first endoscopic treatment for bile leak), a biliary leak located at the common bile duct/common hepatic duct associated with major injury of the bile duct and a high-grade biliary leak proved to be statistically significant predictors of treatment failure of a biliary leak with a combination of biliary sphincterotomy and the placement of a 10-French biliary stent (*P* < 0.01). Multivariate logistic analysis (Table 4) showed that the type of leak, namely a high-grade biliary leak, was the only independent prognostic factor associated with treatment failure (OR = 26.78; 95 % CI = 6.59–108.83; *P* < 0.01).

**Table 2:** Outcomes after endotherapy of postcholecystectomy biliary leaks in 178 patients with a combination of biliary sphincterotomy and the placement of 10-French plastic stent, including adverse events

Outcome	
Clinical success of endotherapy, n (%)	162/178 (91.0)
Cessation of bile output in percutaneous drains, time (days), median (range), mean ± SD	6 (2–9), 5.5 ± 1.4
Duration of stenting, time (days), median (range), mean ± SD	67 (15–86), 47.1 ± 22.4
Number of patients with high-grade leaks treated successfully, n (%)	9/19 (47.4)
Number of patients with adverse events, n (%)	9/178 (5.1)
Stent migration	2/178 (1.1)
Bleeding	2/178 (1.1)
Pancreatitis	5/178 (2.8)

*SD* standard deviation

**Table 3:** Univariate analysis of treatment failure in 178 patients with a combination of biliary sphincterotomy and the placement of a 10-French biliary stent

Factor		Closure of No - n (%)	The leak Yes - n (%)	P
Sex	Female	6 (6,5)	87 (93,5)	0,216
	Male	10 (11,8)	75 (88,2)	
Age	Mean ± SD	61,5 ± 15,6	64,0 ± 15,4	0,542
Site	Cystic duct stump	9 (8,3)	100 (91,7)	0,017*
	CBD/CHD	6 (22,2)	21 (77,8)	
	Luschka	1 (2,4)	41 (97,6)	
Type of leak	High-grade	10 (52,6)	9 (47,4)	0,000**
	Low-grade	6 (3,8)	153 (96,2)	
Interval between surgery and first ERCP	Mean ± SD	6,6 + 3,2	7,6 + 3,8	0,314

*CBD/CHD* Common bile duct/common hepatic duct; *SD* Standard deviation; *ERCP* Endoscopic retrograde cholangiopancreatography

* *P* <0.05; ** *P* < 0.01

**Table 4:** Results of a multivariate logistic model to evaluate independent predictors for failure of the first endoscopic treatment in 178 patients

	OR	*P*	95 % CI for OR	
			Lower	Upper
Age	0,99	0,657	0,95	1,03
Sex - Male	3,40	0,091	0,82	14,00
Leak site – Cystic duct stump	1,79	0,608	0,19	16,47
Leak site - CBD/CHD	3,62	0,319	0,29	45,34
Type of leak – High grade	26,78	0,000*	6,59	108,83
Interval between surgery and first ERCP	0,89	0,161	0,75	1,05

*CI* confidence interval; *OR* odds ratio; *CBD/CHD* common bile duct/common hepatic duct; *ERCP* endoscopic retrograde cholangiopancreatography

* *P* < 0.01

### Rescue endotherapy for refractory biliary leaks

The remaining 16 patients in whom the initial endoscopic treatment was unsuccessful were considered to be clinical failures, and the follow-up ERCP with the removal of the plastic stent confirmed the persistence of a biliary leak. All 16 patients were retreated with the placement of MPSs. At the end of this new endoscopic management, clinical success was obtained in 10/16 patients (62.5 %). In the 10 patients with clinical success, ceased bile output in the percutaneous drains was observed after a median time of 12 days (range, 9–18 days). In this group of 10 successfully treated patients, the median value of the maximum number of stents simultaneously inserted was 3 (range, 2–4), and the median maximum stent diameter was 27 French (range, 20–35.5 French). In the remaining 6 patients without clinical success, the median value for the maximum number of stents simultaneously inserted was 2 (range, 2–2), and the median maximum stent diameter was 19.25 French (range, 18.5–20 French). The univariate analysis of treatment failure with MPSs is shown in Table 5. Of the 6 evaluated variables (sex, age, biliary leak location, type of leak, maximum stent diameter and maximum number of stents simultaneously inserted), a high-grade biliary leak and the use of fewer than 3 plastic stents proved to be statistically significant predictors of treatment failure of a refractory biliary leak with MPSs (*P* < 0.01).

**Table 5:** Univariate analysis of treatment failure with two or more biliary plastic stents (MPSs) used as a rescue endotherapy after failure of the initial endoscopic treatment

		Closure of No - n (%)	The leak Yes - n (%)	*P*
Sex	Female	2 (33,3)	4 (66,7)	0,790
	Male	4 (40)	6 (60)	
Age	Mean ± SD	58,2 + 14,2	63,5 + 16,7	0,480
Leak site	Cystic duct stump	2 (22,2)	7 (77,8)	0,118
	CBD/CHD	4 (66,7)	2 (33,3)	
	Luschka	0 (0)	1 (100)	
Leak type	High-grade	6 (60,0)	4 (40)	0,034*
	Low-grade	0 (0)	6 (100)	
Maximum number of plastic stents inserted	Mean ± SD	2,0 ± 0,0	2,8 ± 0,8	0,023*
Maximum plastic stents diameter reached, French	Mean ± SD	19,5 ± 0,8	24,0 ± 5,7	0,063

*CBD/CHD* common bile duct/common hepatic duct; *SD* standard deviation

* *P* < 0.05

**Fig. 2:**
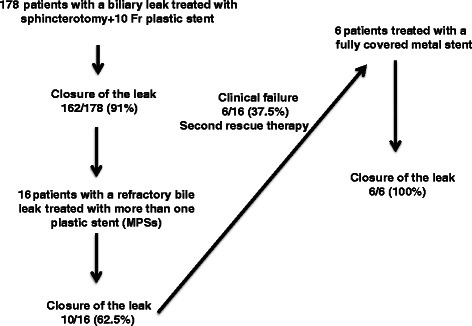
Flow chart of the study

The remaining 6 patients for whom the placement of MPSs was unsuccessful were retreated with the placement of an FCSEMS. At the end of this new endotherapy, leak closure was obtained for all patients. In this group of 6 patients retreated with an FCSEMS, cessation of bile output in the percutaneous drains was observed after a median time period of 6 days (range, 5–9 days), and the metal stents were removed after a median period of 26 days (range, 15–29 days). Metal stents were removed without difficulty and without evidence of de novo biliary strictures at the time of the stent removal. Using different endoscopic management strategies, postcholecystectomy biliary leak closure was achieved in all cases, and none of the patients was referred for surgery (Fig. 2).

### Follow-up and adverse events

Three months after being discharged from the hospital, the patients were evaluated. In total, 151 of 178 patients demonstrated no evidence of a biliary leak, and they had no abnormal liver-associated enzyme values (i.e., aspartate aminotransferase, alanine aminotransferase, alkaline phosphatase and gamma glutamyltransferase) and bilirubin (total and direct). The remaining 27 patients demonstrated no evidence of biliary leakage, but because of biliary stricture, these 27 patients were treated with multiple plastic stents for a median time of 12 months.

Adverse events were divided in ERCP related and stent-related. For the first treatment, complications were observed in a total of 9 of the 178 (5.1 %) patients: mild pancreatitis in 5 of 178 (2.8 %) patients, bleeding in 2 of 178 (1.1 %) patients and stent migration in 2 of 178 (1.1 %) patients. The five cases of pancreatitis were managed conservatively, and all five patients recovered without long-term sequelae. The two cases of bleeding were considered mild and needed no endoscopic treatment to stop the bleeding. Stent migration was observed in two patients at the moment of the stent removal. At that time, the leak was closed, and no further intervention was needed.

For the second treatment in which 16 patients were retreated with MPSs, there were no complications related to ERCP or the stent. Furthermore, for the six patients who were submitted to FCSEMS, there were no cases of pancreatitis, metal stent migration, de novo choledocholithiasis or bile duct strictures found within the FCSEMS or bile duct at the time of the stent removal.

## Discussion

According to the findings of this retrospective, multi-centre study, endotherapy for postcholecystectomy biliary leaks, with a combination of biliary sphincterotomy and the placement of a large-bore plastic stent, is associated with a high success rate (91 %); having a high-grade biliary leak was the only independent prognostic factor associated with treatment failure. The use of MPSs as a rescue therapy for refractory biliary leaks is associated with clinical failures in approximately 40 % of cases, particularly in cases of a high-grade leak or after using fewer than 3 plastic stents. All patients for whom the placement of MPSs failed were retreated with a FCSEMS, resulting in leak closures in all cases, suggesting that refractory biliary leaks should be treated with FCSEMS, particularly in patients with high-grade leaks. Surgery is usually not necessary for managing postcholecystectomy biliary leaks.

Latrogenic injury of the biliary tract wall can occur as a complication of surgical procedures; bile leak was the most commonly reported injury, particularly after cholecystectomy [1, 3, 14]. Furthermore, the advent of laparoscopic cholecystectomy led to an increase in the frequency of biliary leaks [1, 3]. Endoscopy (namely ERCP) is the first line of treatment for such cases. ERCP diagnoses the biliary lesion and significantly decreases the pressure gradient at the ampullary level, thus allowing a preferential flow of bile from the bile duct to the duodenum and leading the defect to seal [1, 3, 5–12]. In 1991, Binmoeller reviewed 77 cases of endoscopically managed postoperative biliary leaks and reported a clinical success in 82 % of the cases [24]. Since that report, data published over a 20-year period (and longer) suggest a success rate of 90 % or better for leak closure [5]. Several methods, namely nasobiliary drains or plastic stents, with or without sphincterotomy and biliary sphincterotomy alone, seem to be equally effective in facilitating the healing of a bile leak, and each method has its own limitations and advantages [5, 8, 25]. In one study, 63 patients with postcholecystectomy biliary were randomized to receive a 7-French stent or a 10-French stent; the clinical success of each strategy was similar, suggesting that the stent size does not affect the outcome of endoscopic intervention [9]. Another group compared biliary stenting alone with biliary stenting plus sphincterotomy and found that the two strategies were equally effective [10]. However, in a retrospective review of 207 patients, one group, found that high-grade leaks were better managed with a combination of stents and sphincterotomy, suggesting that this approach has the highest potential to seal a leak, particularly in more complex or problematic leaks [3]. Furthermore, some authors advocate the use of large bore stents to avoid early clogging and to improve bile flow [5, 14]. Therefore, in the institutions where we work, we follow a standard protocol for biliary leaks that has been used for the last 10 years, namely biliary sphincterotomy and the placement of a large bore (10-Fr) stent. In this study, our clinical success rate was 91 %, which aligns with previously mentioned studies that have reported a clinical success rate between 87.1 % and 100 %, although most of the studies used different endoscopic modalities to seal the leak [1–3, 7, 9, 10, 22, 25]. The prognostic factors for endotherapy have not been systematically addressed in previous reports. In this study, univariate analysis uncovered a biliary leak at the common bile duct/common hepatic duct, which was associated with a major injury of the bile duct and a high-grade biliary leak that proved to be statistically significant predictors of treatment failure, suggesting that more complex leaks are more difficult to heal. Furthermore, a high-grade biliary leak was an independent risk factor, according to multivariate analysis, which might suggest that these types of leaks could be initially managed with a more aggressive strategy.

However, and despite the widespread and success of ERCP, there are still reports of difficult to treat complex leaks requiring multiple re-interventions and, sometimes, surgery [1, 3, 6, 11]. In the last years, two different strategies have emerged as options to treat refractory biliary leaks: placing a temporary FCSEMS or upsizing the existing plastic stent or adding additional plastic stents to further decrease the transpapillary pressure gradient at a lower cost. In this study, after failure of the initial therapy, patients with a refractory leak were submitted to the placement of MPSs avoiding the costs of a FCSEMS. However, in this patient group, the success rate was only 62.5 %, and the use of fewer than 3 plastic stents and a high-grade biliary leak were significant predictors of treatment failure, which might suggest that using MPSs is only clinically effective when at least 3 stents are used and that high-grade leaks should be treated with a FCSEMS. First reported by Baron et al in 2006 [13], the use of a FCSEMS has been shown to be clinically effective in treating refractory biliary leaks at a rate between 90.5 % and 100 % [14, 15, 18, 20, 26]. Some previous studies have reported complications (i.e., migration and “de novo” biliary strictures at the proximal edge of the stent), and the development of these strictures was related with long-term stenting or oversizing (placement of a 10 mm stent in a duct with a smaller diameter) [18, 26, 27]. However, in one study, 17 patients with refractory bile leaks were treated for 30 days or less, with a success rate of 100 % and without complications, suggesting that short-term stenting is efficacious and is not associated with complications [14]. In this study, six patients were submitted to the temporary placement of an FCSEMS for 30 days or less, with success and without complications; this result was most likely related to the short-term stenting. Furthermore, in this study, removing the SEMS was safe, as suggested in a previous multicentre analysis [27]. Together, these finding suggest that the use of MPSs is associated with success in patients with low-grade refractory leaks; although the treatment time is most often longer than one week, the increased hospital stays and costs can be compared with the placement of a FCSEMS in future studies.

The present study has several limitations. First, the retrospective nature limited the number of included patients because some of the identified consecutive patients had incomplete data and were excluded from the analysis. Our data may, therefore, have been subject to selection bias. Another limitation is that there is no direct comparison between the two types of rescue therapy for refractory biliary leaks. We suggest that additional, prospective, randomized studies are warranted in this setting. MPS placement must be compared to temporary placement of a FCSEMS in a large number of patients with refractory biliary leaks to have adequate power to detect minor outcome differences. The strengths of our study are the evaluation of a large number of patients and using a homogenous approach in an attempt to maximize the effect of the initial endotherapy and the evaluation of the available two types of rescue endoscopic management for refractory biliary leaks. To the best of our knowledge, this series is one of the largest available to analyze the prognostic factors for initial and revision treatment failures in detail.

## Conclusion

In conclusion, ERCP using a standard approach with a combination of biliary sphincterotomy and the placement of 10-French plastic stent is associated with a success rate of 91 % in patients with a postcholecystectomy bile leak. However, in our series, there were several failures using MPSs as a strategy for rescue endotherapy, which suggests that refractory biliary leaks should be treated with FCSEMS, particularly in patients with high-grade leaks. In the modern era surgery, is rarely needed to treat postcholecystectomy biliary leaks.

## Abbreviations

ERCP: Endoscopic retrograde cholangiopancreatography
FCSEMS: Fully covered self-expandable metal stent
MPS: Multiple plastic stents
